# Performance Improvement of In-Ga-Zn Oxide Thin-Film Transistors by Excimer Laser Annealing

**DOI:** 10.3390/mi15020225

**Published:** 2024-01-31

**Authors:** Xiaohui Zhang, Yaping Li, Yanwei Li, Xinwang Xie, Longhai Yin

**Affiliations:** 1 Jihua Laboratory, Foshan 528200, China; liyw@jihualab.ac.cn (Y.L.); xiexw@jihualab.ac.cn (X.X.); yinlh@jihualab.ac.cn (L.Y.); 2 School of Materials Science and Engineering, South China University of Technology, Guangzhou 510275, China; liyaping_415@163.com

**Keywords:** thin-film transistors (TFTs), excimer laser annealing, metal-oxide semiconductor devices

## Abstract

We applied excimer laser annealing (ELA) on indium-zinc oxide (IZO) and IZO/indium-gallium-zinc oxide (IGZO) heterojunction thin-film transistors (TFTs) to improve their electrical characteristics. The IZO and IZO/IGZO heterojunction thin films were prepared by the physical vapor deposition method without any other annealing process. The crystalline state and composition of the as-deposited film and the excimer-laser-annealed films were analyzed by X-ray diffraction and X-ray photoelectron spectroscopy. In order to further enhance the electrical performance of TFT, we constructed a dual-heterojunction TFT structure. The results showed that the field-effect mobility could be improved to 9.8 cm^2^/V·s. Surprisingly, the device also possessed good optical stability. The electron accumulation at the a-IZO/HfO, HfO/a-IGZO, and a-IGZO/gate insulator (GI) interfaces confirmed the a-IGZO-channel conduction. The dual-heterojunction TFT with IZO/HfO/a-IGZO-assisted ELA provides a guideline for overcoming the trade-off between high mobility (μ) and positive *V_Th_* control for stable enhancement mode operation with increased *I*_D_.

## 1. Introduction

In recent decades, amorphous metal-oxide semiconductor (AOS) thin-film transistors (TFTs) have attracted extensive attention. Among them, a-InGaZnO (a-IGZO) TFTs are highly favored because they exhibit higher carrier mobility than amorphous silicon TFTs, lower fabricated temperature and better large-area uniformity than those of poly-silicon TFTs, ultralow off-state current, and a steep subthreshold slope [1,2,3,4,5]. With the development of advanced applications, current-driven displays, and back-end-of-line devices, there is a need for AOS TFTs with much higher mobility. Generally speaking, thin films usually require annealing in oxygen or air, which may cause inconvenience in operation and even make it difficult to achieve large-scale annealing. By benefiting from the advantages of laser annealing, rapid annealing of large-area thin films can be conducted [6,7,8].

Improvements to AOS mobility usually include high-temperature annealing, induced crystallization, and the construction of heterojunctions [9,10,11,12,13,14]. According to the reported literature, their mechanisms have basically been clarified. Although the mobility-boosting mechanism in IGZO-based bilayer AOS TFTs has not been thoroughly clarified, it is normally associated with the heterojunction-induced quantum well and also interface defect suppression [15,16,17,18,19,20]. Considering the improvements to film quality by laser annealing and the influence of defects on double-layer AOS channels, we investigated the influence of laser annealing on bilayer AOS devices.

In this work, ELA was processed for postannealing of thin films to improve the electrical performance of devices. An a-IZO single-layer TFT, an a-IZO/a-IGZO heterojunction channel in a single-gate (SG) TFT, and an a-IZO/HfO/a-IGZO trilayer in a dual-gate (DG) TFT were controlled with a bottom-gate (BG) TFT. We attempted to analyze and compare the distinct dependences of the improvement mechanisms of laser annealing and assisted double-gate structures on mobility and stability and reveal the locations and formation mechanisms of carrier transport paths in the AOS heterojunction channel [21,22,23,24,25,26].

## 2. Materials and Methods

Film fabrication: A heavily doped p-Si wafer (gate electrode) with a thickness of 300 nm and a thermally grown SiO_2_ dielectric layer was employed as the substrate. First, the Si/SiO_2_ substrates were ultrasonically cleaned in acetone, alcohol, and deionized water sequentially and then blow-dried with nitrogen. Second, on the Si/SiO_2_ substrates, IZO (10 nm), HfO_x_ (2 nm), and a-IGZO (10 nm) were sputtered in a mixed atmosphere of 10 sccm argon (Ar) and 2 sccm oxygen (O_2_) using direct sputtering of physical vapor deposition. ITO as a source-drain (S/D) electrode was prepared using radio-frequency sputtering. A relatively low power of 100 W was used to achieve a moderate deposition rate and also to suppress incidental bombardment damage to the underneath layer.

TFT fabrication: The AOS TFTs were fabricated in the bottom-gate structure on Si/SiO_2_ substrates. The active layers were made of a 10 nm thick a-IZO single-layer TFT, an a-IZO (10 nm)/a-IGZO (10 nm) dual-layer TFT, and an a-IZO (10 nm)/HfO (2 nm)/a-IGZO (10 nm) trilayer TFT. On the active layers, 100 nm thick sputtered InSnO (ITO) was patterned in DC mode using a mask, forming S/D electrodes with 20 μm channel length and 100 μm channel width. 

**Figure 1:**
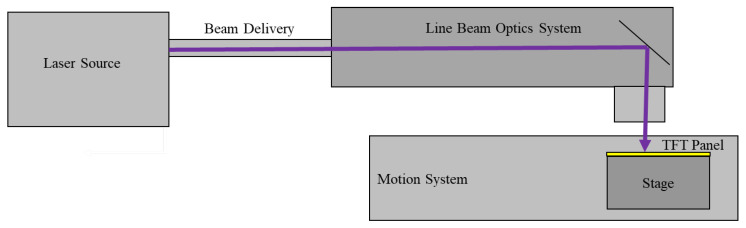
Schematic diagram of the XeCl excimer laser annealing system.

Excimer laser annealing system: This consisted of a laser source of XeCl excimer laser; 308 nm wavelength; max. pulse energy of 1000 mJ; pulse duration of 24 ns ± 4 ns; max. laser repeat rate of 600 Hz; energy stability less than 1%; optics system laser line beam size of 750 (0–750 mm adjustable) × 0.25 mm; laser beam focus range within ±100 µm; max. energy density at substrate of 340 mJ/cm^2^; homogeneity of short axis less than 4%; homogeneity of long axis less than 1.8%; stage moving resolution of 0.1 μm; stage straightness less than 5 μm; and stage max. velocity of 400 mm/s (0–400 mm/s adjustable). A schematic diagram of the XeCl excimer laser annealing system is shown in Figure 1.

**Figure 2:**
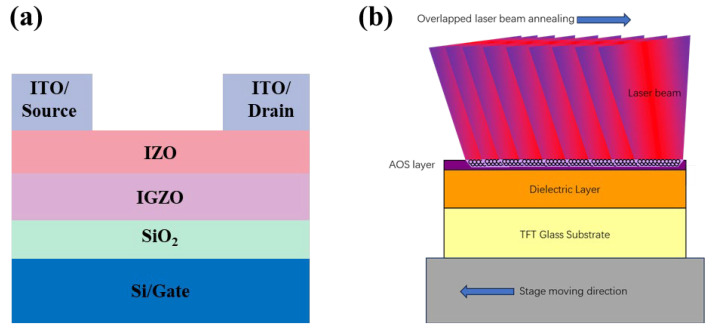
(**a**) Structure of a dual-layer TFT device. (**b**) Schematic diagram of the annealing process by XeCl excimer overlapping laser beams.

Figure 2a presents a picture to clearly exhibit the TFT structure of dual layers in this study. From this, we can see that the TFT device used Si as the substrate and acted as the gate electrode, with SiO_2_ acting as the gate dielectric layer. The subsequent heterojunction was grown on SiO_2_ by the PVD method to provide an active layer, forming a conductive channel for TFT. ITO was finally grown on the active layer as a source and drain electrode. Details of the sedimentary conditions can be found in the experimental section of the text. The schematic diagram of the annealing process of the XeCl excimer laser is shown in Figure 2. The linear laser beam annealed horizontally along the thin film via the stage, moving with the TFT substrate. Due to the controllable stage moving speed, fast TFT AOS surface annealing could be achieved, and the scanning range could also be controlled to achieve large-area surface annealing from the laser line beam size, which was 0–750 mm adjustable. The laser energy density can be adjusted to achieve the best TFT performance based on different film properties. Therefore, by adjusting the stage moving speed, laser line length, laser energy, laser repeat rate, etc., fast and large-scale annealing could be achieved.

## 3. Results and Discussion

**Figure 3:**
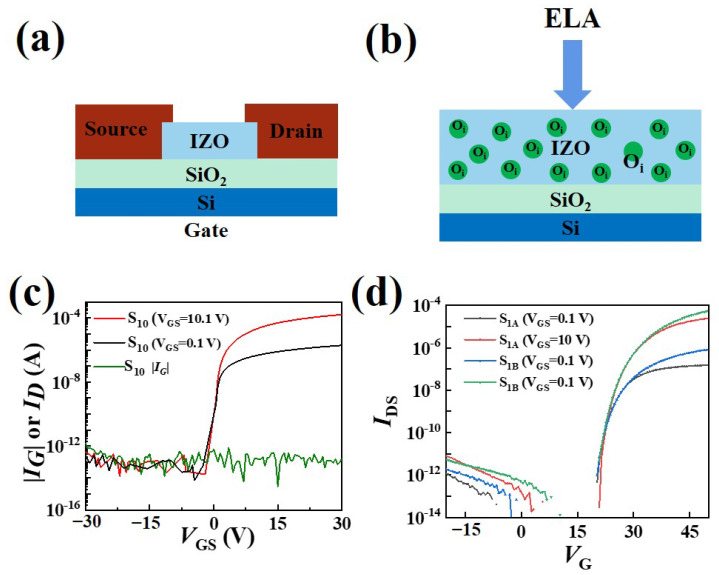
(**a**) Diagram of the TFT device. (**b**) Principle of ELA. (**c**) Transfer curve of the single IZO film (*V*_DS_ = 0.1 and 10.1 V). (**d**) Transfer curve of the IZO film annealed at different laser intensities (S_1A_ curves with 200 mJ/cm^2^ and S_1B_ curves with 250 mJ/cm^2^).

The TFT with single-active-layer IZO was successfully fabricated. Figure 3a shows a simplified diagram of the single-active-layer IZO TFT device, while Figure 3b shows the schematic diagram of the principle of laser annealing. Figure 3c shows the transfer (*I_ds_*-*V_gs_*) curves of the TFT with single-active-layer IZO without ELA (labeled as S_10_). The thickness of the IZO layer was 10 nm. The TFT with single-active-layer IZO exhibited a mobility of 7.65 cm^2^/V·s, an acceptable threshold voltage (*V_th_*, linear fitting based on *I*_DS_^1/2^-*V*_GS_ curve) of 1.4 V, a subthreshold swing (*SS*, extracted from the linear portion of the log(*I_DS_*) versus *V_GS_*, *SS* = d*V_GS_*/dlog*I_DS_*) of 130 mV/decade, and a low off-current below 1 pA. Unfortunately, it exhibited an unclear pinch-off. As we all know, unclear pinch-off often slows down the switching speed of the TFT, which is not conducive to the implementation of fast response devices. Then, ELA was used for annealing treatment with laser energies of 200 and 250 mJ/cm^2^. Figure 3d shows the transfer curve of the IZO single-layer TFT after laser annealing at 200 and 250 mJ/cm^2^ laser energy (labeled as S_1A_ and S_1B_, respectively). When ELA was applied, the TFT exhibited significant changes, such as field effect mobility of 0.69 and 3.45 cm^2^/Vs, *Vth* of 23.48 and 25.27 V, *SS* of 493 and 421 mV/decade at laser intensities of 200 and 250 mJ/cm^2^, respectively. Here, the calculation of mobility was based on the following formula:

$$I_{DS}=\frac{W\mu_{sat}C_{i}}{2L}{\left(V_{G}-V_{T}\right)}^{2}$$

In the formula, *I_DS_* is the current between the source and the drain electrode; *μ_sat_* is the carrier mobility in the saturated region; *C_i_* is the capacitance; *V_G_* is the voltage applied between the source and the gate; *V_T_* is the threshold; and *W* and *L* are the width and length of conductive channels, respectively. Fortunately, the IZO single-layer TFT after ELA treatment exhibited a clear pinch-off. This phenomenon is attributed to the laser annealing process and may be due to a large number of M-O bond breaks during the ELA process, resulting in the generation of many interstitial oxygen vacancy defects in the IZO film that could capture electrons, ultimately leading to a significant positive threshold voltage of the device. This is similar to the pure oxygen annealing reported in the literature, where the oxygen partial pressure is reduced during the annealing process (such as by introducing argon gas), the gap oxygen defects are reduced, and the device *V_th_* becomes negative accordingly [27,28,29,30]. 

**Figure 4:**
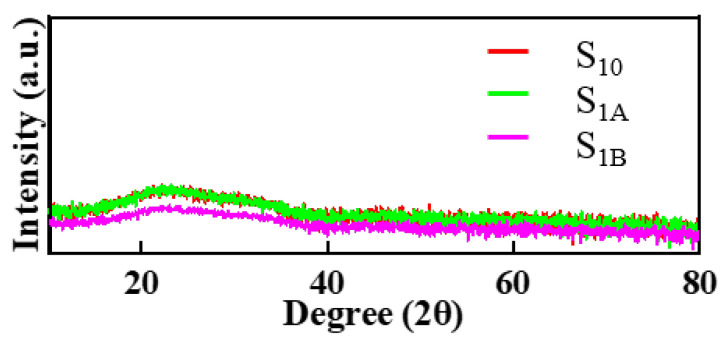
XRD of samples S_10_, S_1A_, and S_1B_.

We analyzed whether the crystalline state of the thin film changed before and after ELA using X-ray diffraction (XRD). The XRD spectrum in Figure 4 shows that there was no change in the crystalline state of the thin film before and after ELA, whether annealed at a laser intensity of 200 or 250 mJ/cm^2^. In a word, the thin films of the three devices were all amorphous. Then, we attempted to verify the rationality of the above conjecture about oxygen content by examining the O 1S content of the thin film.

**Figure 5:**
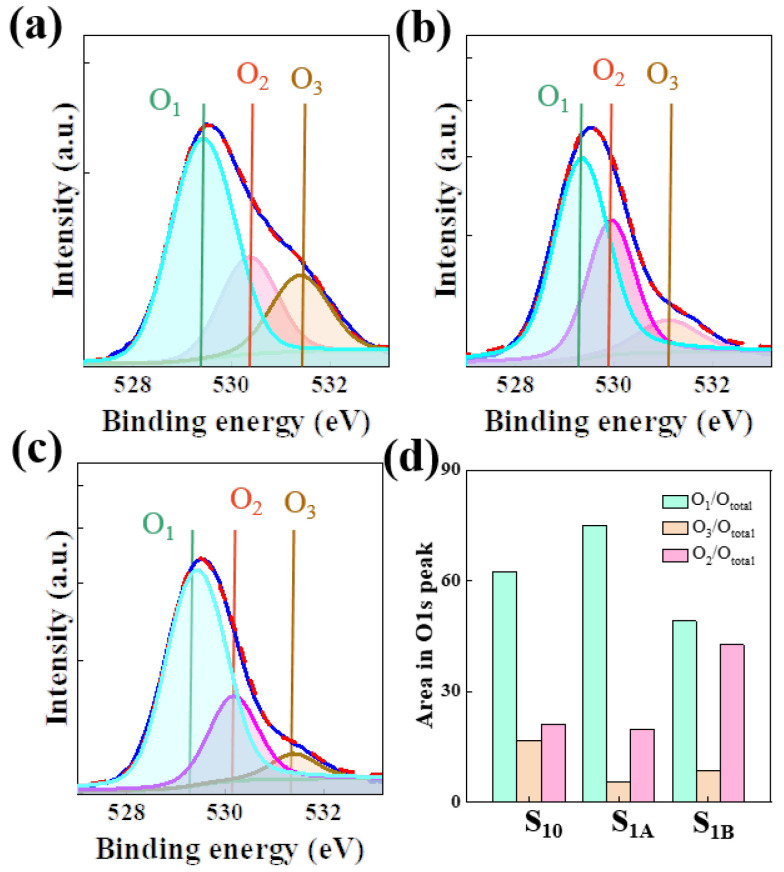
XPS spectra of O1s patterns of the a-IGZO active layers deposited by PVD. (**a**) As-deposited. (**b**) Laser annealed at a power of 200 mJ/cm^2^. (**c**) Laser annealed at a power of 250 mJ/cm^2^. (**d**) Area in the O1s peak of S_10_, S_1A_, and S_1B_.

Figure 5 shows the results of an X-ray photoelectron spectroscopy (XPS) analysis of the O1s peaks of the as-deposited a-IZO active layer without ELA processing and the a-IZO active layers with ELA processing at laser intensities of 200 and 250 mJ/cm^2^. As shown in Figure 5a, 5b for the a-IZO active layer without laser annealing and laser annealing at a laser intensity of 200 mJ/cm^2^, respectively, the ratio of the lattice oxygen peak (O_1_) to all O1s peaks increased from 62.75% to 74.9%, but the ratio of the hydroxide peak (O_3_) to all O1s peaks decreased from 16.5% to 5.3%. These results indicate that the oxygen vacancy concentration in the a-IZO thin film increased due to laser annealing, and this created an effect of an increase in electron concentration. Here, the oxygen vacancies played the role of doubly charged donors according to the relationship in the Kröger–Vink notation (V_ox_→V_o_ + 2e^−^) [31,32,33,34,35]. As shown in Figure 5c, when the annealed laser intensity increased to 250 mJ/cm^2^ for the a-IZO active layer, the O_1_/O_total_ value decreased to approximately 49.1%, while the O_3_/O_total_ value decreased to approximately 8.3%. When the O_1_ peak becomes smaller and the oxygen vacancy peak (O_2_) peak becomes larger, the metal–oxygen bond is not restored properly, while the oxygen vacancies remain and the electron concentration increases. Therefore, the conductivity increases, and the saturation current should be appropriately increased. Therefore, our proposed fast laser annealing method will be effective for adjusting the performance of TFTs.

Next, we introduced the IZO/IGZO double layers as an active layer with ELA processed at laser intensities of 200 and 250 mJ/cm^2^ to improve the performance of the device (labeled as S_2A_ and S_2B_, respectively). Also, we examined the *V_th_* stability of the TFTs during the gate voltage stress.

**Figure 6:**
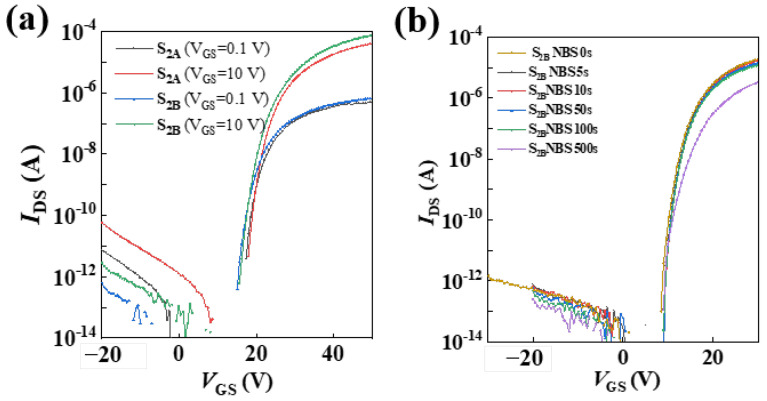
(**a**) Transfer curve of the IZO/IGZO heterojunction TFT annealed at a laser power of 250 mJ/cm^2^. (**b**) Negative bias stability under illumination of the IZO/IGZO heterojunction TFT (250 lux white LED).

It was found that the threshold voltage of the device decreased to 21.49 and 19.98 V when annealed at laser intensities of 200 and 250 mJ/cm^2^, respectively, as shown in Figure 6a. The mobility of the device did not show a significant improvement, with 2.04 at 200 mJ/cm^2^ and 1.91 at 250 mJ/cm^2^, respectively. As we all know, the IZO layer for high mobility has a lower resistance than the IGZO layer. The dual active layer of IZO/GIZO reduced the mobility of IZO, and both IZO and IGZO acted as conductive channels, resulting in a decrease in the overall mobility of the device [36,37,38]. Next, negative bias instability (NBS, *V_GS-stre_* = 30 V, and *V_DS_* = 10 V) under illumination at 250 lux of the heterojunction devices was tested. Surprisingly, the IZO/IGZO heterojunction device annealed by a 250 mJ/cm^2^ laser exhibited good optical stability, as shown in Figure 6b, which indicates that the *V_th_* stability of the TFT with a double active layer was caused by the IGZO channel layer. At this point, IZO/IGZO heterojunction-assisted ELA did not have a significant effect on reducing the threshold of the device but was excellent at assisting negative bias illumination stability. 

**Figure 7:**
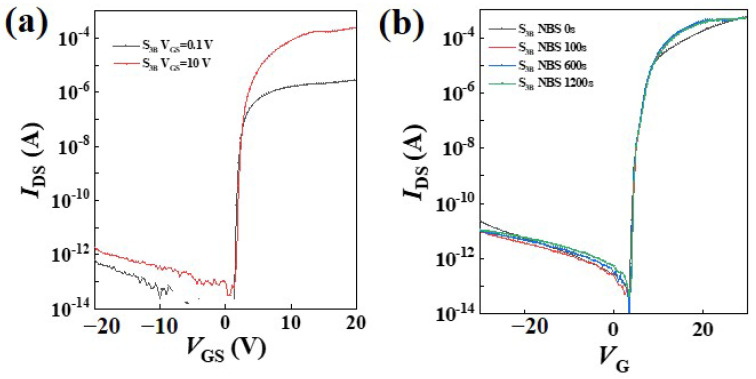
(**a**) Transfer curve of IZO/HfO/IGZO dual-heterojunction TFT annealed at a laser intensity of 250 mJ/cm^2^. (**b**) Negative bias stability under illumination of the IZO/HfO/IGZO heterojunction TFT (275 lux white LED).

In order to improve the mobility and other performance metrics of the device without reducing the negative bias photostability, a dual-heterojunction structure (IZO/HFO_x_/IGZO) was constructed and annealed at a laser intensity of 250 mJ/cm^2^ (labeled as S_3B_). As shown in Figure 7a, the dual-heterojunction structure under 250 mJ/cm^2^ laser power showed a mobility of 9.8 cm^2^/V·s, *V_th_* of 1.86 V, and *SS* of 97 mV/dec. Negative bias instability (*NBS*, *V_GS-stre_* = 30 V, and *V_DS_* = 10 V) under illumination at 250 lux was also tested. The dual-heterojunction TFT showed a *V_th_* shift of less than 0.6 V, and these results were similar to those of the IZO/IGZO heterojunction TFT, as shown in Figure 7b. These results indicate that the *V_th_* stability of the dual-heterojunction TFT was caused by the IGZO channel layer and had high mobility due to its dual-electron channels. 

Table 1 was compiled to visually distinguish the impact of ELA and heterostructure construction on the performance of TFT devices. 

## 4. Conclusions

This work created a-IZO, IZO/IGZO, and IZO/HfO_x_/IGZO TFTs by applying ELA for 200 and 250 mJ/cm^2^. To analyze the effect of ELA on a-IZO, and IZO/IGZO, IZO/Hf_x_/IGZO TFTs, XRD and XPS were carried out to analyze the properties of the films. In this study, the IZO/Hf_x_/IGZO TFTs to which a 250 mJ/cm^2^ laser was applied had an electron mobility of 9.8 cm^2^/V·s, an on-off-current ratio of 9.6 × 10^8^, and an *SS* value of 0.097 V/dec, which was a great improvement over the a-IZO TFT without the laser applied and the IZO/IGZO heterojunction devices processed by ELA. Therefore, we propose an effective method of ELA combined with a dual-heterojunction structure to improve both the mobility and stability of TFTs.

## Figures and Tables

**Table 1:** The electrical parameters of the TFTs with various active layers. The mobility (µ), *SS*, and *V_th_* are summarized.

Chanel	Annealed	*V_th_* (V)	*SS* (mV/dec)	μ (cm^2^/V·S)
S10	As-deposited	1.4	128	7.65
S1A	ELA at 200 mJ/cm^2^	23.48	493	0.69
S1B	ELA at 250 mJ/cm^2^	25.27	421	3.45
S2A	ELA at 200 mJ/cm^2^	21.49	590	2.04
S2B	ELA at 250 mJ/cm^2^	19.98	667	1.91
S3B	ELA at 250 mJ/cm^2^	1.86	97	9.8

S10: Sample of the single-layer IZO TFT without ELA; S1A: sample of the single-layer IZO TFT with ELA at laser intensity of 200 mJ/cm^2^; S1B: sample of the single-layer IZO TFT with ELA at laser intensity of 250 mJ/cm^2^; S2A: sample of the IZO/IGZO TFT with ELA at laser intensity of 200 mJ/cm^2^; S2B: sample of the IZO/IGZO TFT with ELA at laser intensity of 250 mJ/cm^2^; S3B: sample of the single-layer IZO/HfO/IGZO TFT with ELA at laser intensity of 200 mJ/cm^2^.

## Data Availability

The data generated in the present study are available from the corresponding author upon reasonable request.
